# Perceived exertion, comfort and working technique in professional computer users and associations with the incidence of neck and upper extremity symptoms

**DOI:** 10.1186/1471-2474-13-38

**Published:** 2012-03-21

**Authors:** Agneta Lindegård, Jens Wahlström, Mats Hagberg, Rebecka Vilhelmsson, Allan Toomingas, Ewa Wigaeus Tornqvist

**Affiliations:** 1 Institute of Stress Medicine, Göteborg, Sweden; 2 Department of Public Health & Clinical Medicine, Occupational and Environmental Medicine, Umeå University, Umeå, Sweden; 3 Occupational and Environmental Medicine, Sahlgrenska Academy and University hospital, Göteborg, Sweden; 4 Clinical Information Science AstraZeneca R & D, Mölndal, Sweden; 5 Karolinska Institutet, Institute of Environmental Medicine, Stockholm, Sweden; 6 School of Health Sciences, Jönköping University, Jönköping, Sweden

**Keywords:** Computer work, Perceived exertion, Perceived comfort, Working technique, neck and upper extremity disorders

## Abstract

**Background:**

The aim of this study was to investigate whether perceived exertion, perceived comfort and working technique is associated with the incidence of neck and upper extremity symptoms among professional computer users.

**Methods:**

At baseline a self-administered questionnaire was distributed to 853 participants from 46 different work sites (382 men and 471 women) who, at baseline, had been free from neck and upper extremity symptoms during the preceding month. Work-related exposures, individual factors, and symptoms from the neck and upper extremities were assessed. Observations of working technique were performed by ergonomists using an ergonomic checklist. Incidence data were collected by means of 10 monthly questionnaires, asking for information on the occurrence of neck, shoulder and arm/hand symptoms. Perceived exertion was rated on a modified Borg RPE scale ranging from 0 (very, very light) to 14 (very, very strenuous). Perceived comfort was rated on a 9-point scale ranging from -4 (very, very poor) to +4 (very, very good) in relation to the chair, computer screen, keyboard, and computer mouse.

**Results:**

The median follow up time was 10.3 months. The incidence of symptoms from the neck, shoulders and arm/hands were 50, 24 and 34 cases per 100 person years, respectively.

Higher perceived exertion in the neck, shoulder or arm/hands was associated with an increased risk of developing symptoms in the corresponding body region. Moreover, a dose-response relationship between the level of exertion and the risk of developing symptoms was recorded for all three regions. There was an association between low comfort and an increased risk for neck symptoms, but not for shoulder and arm/hand symptoms, although a trend towards such an association (not statistically significant) could be seen. Working technique was, in this study, not associated with the risk of developing symptoms in any of the investigated body regions.

**Conclusion:**

There was a strong association between high perceived exertion and the development of neck, shoulder, and arm/hand symptoms. Moreover, there was an association between poor perceived comfort and neck pain. Surveillance of computer users may include perceived exertion and comfort to target individuals at risk for neck and upper extremity symptoms.

## Background

Musculoskeletal pains and aches are prevalent in the general population in many countries [1,2]. Within the European Union (EU) a 12 month prevalence of 23% has been reported for work related musculoskeletal disorders [3]. In Sweden the prevalence of these disorders has decreased slightly during recent years but it still constitutes one of the major risk factors leading to long term sick leave [4]. Apart from individual suffering and a decrease in the quality of life, these disorders place a heavy economic burden on society due to costs connected to long term sick leave, poorer work performance and reduced productivity [5,6].

The causes of work related neck and upper extremity symptoms continue to be insufficiently understood. Both cross sectional and longitudinal studies have suggested, however, that factors related to the individual (e.g. age and gender), working technique, working postures, muscular rest and perceived muscle tension as well as factors related to the work place or work organization, such as workplace layout, repetitive and constrained work and psychosocial working conditions, may be potential risk factors [7-12]. Similar risk factors have been found for computer work [13-18]. For instance, poor working technique or work style, as described by Feuerstein and coworkers [13], has been shown to be associated with an increased risk of developing symptoms indicative of neck and upper extremity disorders [19-21]. Over the years, several models have been developed in an attempt to identify and explain possible links between different exposures, early signs of incipient musculoskeletal pain conditions and more manifest musculoskeletal outcome. One of these models is the ecological model of musculoskeletal disorders in office work, presented by Sauter and Swansson in 1996 [22]. A modified version of this model, specifically targeting computer work has been proposed by Wahlström in 2005 [23]. In this model, biomechanical factors, psychosocial factors and mental stress, modified by individual factors, may manifest as different detect sensations (early signs) preceding more manifest neck and upper extremity symptoms. The model underlying the present study is that high perceived exertion and low perceived comfort during computer work, might be such early signs and therefore important to identify in order to target individuals at risk of developing severe and long lasting musculoskeletal symptoms/conditions. Consistent with this hypothesis, an earlier cross-sectional study among call center workers has shown an association between poor work place comfort (including lighting conditions, noise, temperature etc) and a higher prevalence of neck and upper extremity symptoms [24]. Regarding perceived exertion there are indications that high perceived exertion may be a crude general measure of an elevated risk of neck and upper extremity pain among computer workers [25].

Hence, the main aim of this longitudinal cohort study was to investigate:

1. Whether perceived exertion and perceived comfort, respectively, are associated with the incidence of neck and upper extremity symptoms.

2. Whether observed working technique is associated with the incidence of neck and upper extremity symptoms.

## Methods

The study described herein is a prospective cohort study among professional computer users, with an observation period of 10 months. At baseline a self-administered questionnaire was used to assess work-related exposures, individual factors, and symptoms from the neck and upper extremities.

In addition, observations of working technique were performed by ergonomists using an ergonomic checklist designed for the assessment of computer work exposures [26].

The study was approved by the local ethics committee at the Karolinska Institutet and the regional ethics committee at Gothenburg University.

### Study population

**Figure 1:**
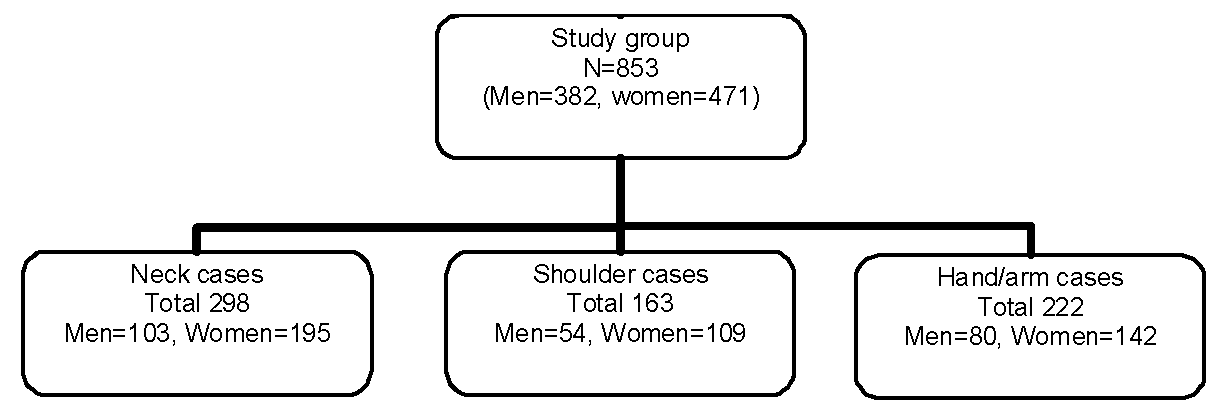
**Chart showing the study population, participants at baseline, and number of cases affecting the investigated body regions for men and women**.

The participants were recruited by ergonomists employed by different Occupational Health Care Centers. The initial study population included 1529 participants. The baseline questionnaire was answered by 1283 subjects. The study group consisted of 853 participants from 46 different work places, representing a great variety of professionals (librarians, engineers, graphic designers, receptionists, secretaries, journalists, researchers, insurance officers and call center personnel) from both the private and the public sector (382 men and 471 women) who, at baseline, had been free from neck and upper extremity symptoms during the preceding month (Figure 1). The mean age of the men was 42 years and 44 years for the women. The self-reported time spent on computer work was, on average, 3.7 hours/day for the men and 3.8 hours/day for the women (Table 1). During the remaining working time the participants, depending of their professions, performed ordinary office work including reading, writing, sorting, calculating, making telephone calls, attending meetings etc. Thus, there was a great variability in self-reported time with computer work between participants.

**Table 1:** Baseline characteristics of participants, presented as mean values with range within brackets

	Men (n = 382)	Women (n = 471)
Age (y)	42.4 (19.6-65.3)	44.5 (21.4-64.7)
Height (m)	1.81 (1.57-2.00)	1.67 (1.44-1.87)
Weight (kg)	81.1 (51-135)	65.2 (45-110)
Smokers (%)	11%	18%
Computer work (h/d)	3.7 (0.2-8.3)	3.8 (0.2-10.0)
Job tenure (y)	9.7 (0.1-42)	13.2 (0.2-40)

### Follow-up

Incidence data were collected by means of 10 postal monthly questionnaires, asking for information on the occurrence of neck and upper extremity symptoms. The questions referred to the time period since completion of the preceding questionnaire, which was approximately one month but could have been longer due to vacations or other reasons for absence. If a follow-up questionnaire was not returned before the next one was available, the time frame used for reporting symptoms covered the whole period since the previous questionnaire was answered, i.e. approximately two months. If two consecutive questionnaires were missing, the calculated person-time connected to that participant was closed when the last questionnaire was answered.

### Assessment of symptoms

**Figure 2:**
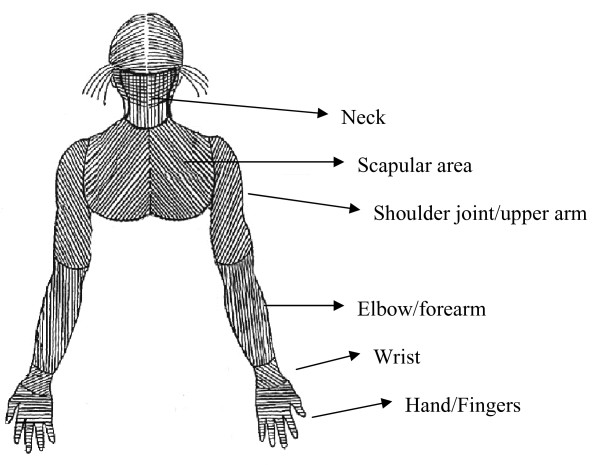
**Showing the body regions considered for rating of perceived exertion in the present study**.

The monthly questionnaires asked if the participants had experienced symptoms of pain or ache during the preceding month, in any of the following body regions: neck and right and left scapular areas, shoulder joint/upper arms, elbow/forearms, wrists, and hands/fingers (see Figure 2). If they reported any symptoms the duration (number of days) in the respective regions were requested.

### Ratings of perceived exertion

Perceived exertion after a typical work day was rated, in the baseline questionnaire, on a modified Borg RPE scale ranging from 0 (very, very light) to 14 (very, very strenuous) for each of the 11 body regions under examination (Figure 2) [27].

### Ratings of comfort

Perceived comfort was rated, in the baseline questionnaire, on a 9-point scale ranging from -4 (very, very poor) to +4 (very, very good) in relation to the chair, computer screen, keyboard, and computer mouse [28].

### Observation of working technique

Working technique was observed at baseline by ergonomists (n = 32) according to the checklist. They were trained in using the checklist accurately and in a standardized manner during seminars before the "real life" observations were made. The participants were observed at their work places during ordinary computer work (observation time ranging from 10-15 minutes for each subject). The observations were characterized on the basis of eight variables: 1) use of forearm support during keyboard work; 2) use of forearm support during computer mouse work; 3) elevation of the shoulders during keyboard work; 4) elevation of the shoulders during computer mouse work; 5) sitting in a tense position when using the keyboard; 6) sitting in a tense position when using the computer mouse; 7) range of movement when performing computer mouse work; 8) speed and/or jerkiness of the computer mouse movements.

### Data treatment and analysis

Symptoms from the 11 different body regions were combined into symptoms from three major regions: a) the neck and the scapular region (hereafter called the neck region); b) shoulder and upper arms (hereafter called the shoulder region); c) elbows/forearms, wrists and hands/fingers (hereafter called the arm/hand region). A "case" was defined as a participant who reported maximum 2 days with symptoms during the preceding month in all body regions ("symptom free") at baseline and who later, at any follow up occasion, reported symptoms lasting ≥ 3 days during the preceding month in the specific body region, i.e. a, b or c. The incidence rate was calculated as the frequency of "new" cases divided by the total person-time-at risk. Subjects contributed with person-time corresponding to the period between the dates of the baseline questionnaire and the date when they became a case or the date when they completed their last questionnaire (non-cases).

To evaluate perceived exertion, a sum score was calculated for the neck, shoulder, and arm/hand region, respectively. The sum score for each body region was then divided by the number of areas included for each body region in order to obtain a mean value. Subjects were classified into three groups, with 0-4 (less than relatively light exertion) as the reference group, 5-7 (relatively light - somewhat strenuous) as the medium exertion group, and ≥8 (strenuous or very strenuous) as the high exertion group.

For comfort, a sum score for comfort was calculated for the included items and divided by the number of items included in the score in order to obtain a mean value. Subjects were then classified into three groups, where -4 to -1 was classified as poor comfort, 0 to +2 as acceptable or medium comfort and ≥ 3 as good comfort (the reference group).

The working technique scores for each of the eight variables included were combined into an overall score ranging from 1-22 [25]. Subjects scoring ≥14 were classified as having a good working technique, those scoring 12-13 as having an acceptable working technique, and those scoring < 12 as having a poor working technique [29].

Incidence rate ratios (relative risks, RR) with 95% confidence intervals (95% CI) for symptoms in the neck, shoulder, and arm/hand region were calculated using Cox proportional hazard models in the software JMP version 5.0.1 and Proc Phreg (SAS v.9.0) and adjusted for age, sex and time spent undertaking computer work. The rationale behind controlling for the computer use time was partly the great variability in the amount of time spent undertaking computer work in the study population and partly the assumption that computer time might co-vary with both the perceived exertion and comfort and musculoskeletal symptoms and thus a potential confounder as described by Rothman [30].

## Results

The median follow up time was 10.3 months (interquartile range 4.1-11.2 months). The incidence rate of symptoms from the neck, shoulders and arm/hands were 50, 24 and 34 cases per 100 person years, respectively.

**Figure 3:**
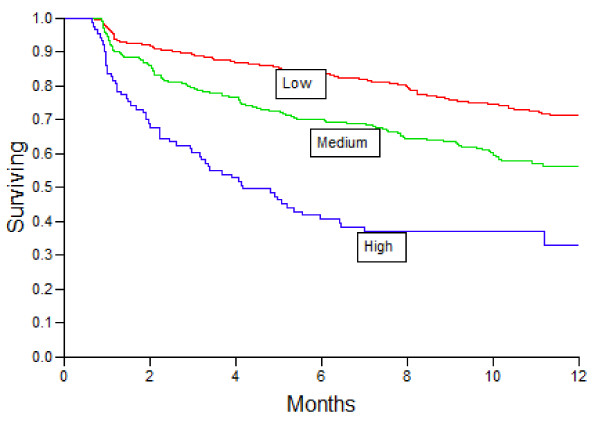
**Kaplan-Meier survival curve for ratings of perceived exertion in the neck (unadjusted) for low (0-4), medium (5-7) and high (8-14) perceived exertion groups**.

Our results showed that higher perceived exertion in the neck, shoulder or arm/hands was associated with an increased risk of developing symptoms in the corresponding body region (Table 2). Moreover, a dose-response relationship between the level of perceived exertion and the risk of developing symptoms was recorded for all three regions. In addition, participants in the high exertion group reported earlier onset of symptoms than did subjects reporting medium or low exertion in the neck (Figure 3). Regarding perceived comfort, there was an association between low comfort and an increased risk for neck symptoms, but not for shoulder and arm/hand symptoms, although a trend towards such an association (not statistically significant) could be seen (Table 2).

**Table 2:** Relative risks (RR) with 95% confidence intervals (95% CI) for neck and upper extremity symptoms in relation to perceived exertion, working technique score and comfort

	Neck		Shoulder		Arm/hand	
	
	Cases/Non cases	RR (95% CI)	Cases/Non cases	RR (95% CI)	Cases/Non cases	RR (95% CI)
**Perceived Exertion**						
*Neck*	298/553					
Low (0-4)	128/351	1				
Medium (5-7)	111/166	**1.70 (1.32-2.20)**				
High (8-14)	59/36	**3.20 (2.31-4.38)**				
						
*Shoulder*			163/689			
Low (0-4)			88/521	1		
Medium (5-7)			50/147	**1.96 (1.38 -2.77)**		
High (8-14)			25/21	**5.49 (3.41 -8.51)**		
						
*Arm/hand*					220/631	
Low (0-4)					154/528	1
Medium (5-7)					56/97	**1.86 (1.36-2.51)**
High (8-14)					10/6	**4.41 (2.16 -8.03)**
						
**Working Technique**	245/435		130/550		176/504	
Good (> 14)	162/303	1	92/373	1	121/344	1
Acceptable (13-14)	55/88	1.06 (0.77-1.43)	27/116	0.89 (0.57-1.35)	37/106	0.91 (0.62-1.31)
Poor (< 13)	28/44	1.03 (0.67-1.51)	11/61	0.73 (0.37-1.30)	18/54	0.90 (0.53-1.44)
						
**Comfort**	298/551		163/686		222/627	
Good (≥3)	55/132	1	31/156	1	44/143	1
Medium (1-3)	190/345	**1.41 (1.05-1.92)**	105/430	1.37 (0.92-2.09)	140/395	1.21 (0.86-1.72)
Poor (-4-0)	53/74	**1.88 (1.28-2.76)**	27/100	1.62 (0.95-2.73)	38/89	1.53 (0.98-2.38)

Adjusted for age, sex and duration of daily computer work. Bold figures represent statistically significant results

Working technique was in this study not associated with the risk of developing symptoms in any of the body regions considered (Table 2).

## Discussion

### Perceived exertion and comfort

The main result of this study was that high perceived exertion in the neck, shoulder, and arm/hand during computer work is strongly associated with an increased risk of developing musculoskeletal symptoms in the corresponding body region. The results confirm the model suggesting that high perceived exertion is an early sign preceding musculoskeletal symptoms in the neck and upper extremities. Moreover, low perceived comfort is similarly, an early sign associated with an increased risk of neck pain but not of pain in the shoulder and hand/arm region. The clinical significance of the results from this study is that perceived high exertion and/or low comfort could be used in surveys in order to detect computer users at risk for neck and upper extremity musculoskeletal symptoms. Furthermore, in line with the previously identified association between high perceived muscle tension and neck pain [31] presented in the proposed model for computer work and musculoskeletal disorders by Wahlström [23], high perceived exertion and low perceived comfort should be regarded as independent risk factors for future neck and upper extremity symptoms. Similar to the above mentioned study, the risk estimate for perceived exertion in the current study remained significant when potential confounders such as age, sex and computer use time were controlled for.

To our knowledge most studies performed in this field have focused on the relationship between exposures and exertion and/or comfort [32] or on different exposures and symptoms [16,33,34]. However, another publication from the present cohort and a study investigating musculoskeletal symptoms among call-center workers have, congruent with the results from our study, reported a relationship between poor perceived overall comfort and musculoskeletal symptoms [17,24]. In the study by Tornqvist et al there was a two-fold increased risk of developing neck symptoms among computer workers perceiving low overall comfort [17]. Both the above mentioned studies also found an association between overall perceived comfort and shoulder and arm/hand symptoms, respectively, inconsistent with the lack of association seen in our study. However, this inconsistency might partly be explained by the much broader definition of overall comfort used in the cited studies were also lighting conditions, noise, ventilation and temperature were included.

The validity of subjective ratings has previously been questioned for a number of reasons [35,36]. However, the modified Borg scale (RPE 0-14) [27] used in this study has been used frequently to investigate perceived exertion during both heavy physical work and under more sedentary working conditions such as office work and computer work [28,29,37-39]. With respect to the comfort scale a clear dose-response relationship between ratings of comfort and symptoms in the neck and upper extremities has been found in a cross sectional study of call centre workers, where comfort was recorded in questionnaires similar to those used in this study [24].

### Working technique

In this study, working technique was not associated with the incidence of neck/and upper extremity symptoms, although earlier cross-sectional studies among computer users have indicated such an association [20,21,40]. Likewise, a study on working technique during text editing tasks on mobile phones has indicated differences in working technique between subjects with and without symptoms [41]. One reason for the inconsistency between these studies and our study could be the way working technique is defined. In this study the definition was solely linked to physical factors (forearm support, computer mouse movements, sitting in a tense position etc), while the studies suggesting a positive relationship used a broader definition of working technique including both physical, psychological and behavioral aspects.

#### General discussion

A recent review evaluating the effects of office ergonomic interventions as a secondary preventive action for workers with musculoskeletal disorders concluded that most outcomes were focused on improved comfort among office workers and that the evidence for the effectiveness of these interventions ranged from insufficient to moderate and that more objective measures were needed [42]. Even though perceived exertion and comfort could not be considered as objective measurements, this study provides support for the model that perceived exertion and comfort are feasible markers in surveys targeting individuals at risk of developing neck and upper extremity disorders. Finally, it could be argued that perceived exertion and perceived comfort might just reflect the exposure brought about by for example poor working postures, but this view is contradicted by the fact that another study exploring potential associations between workload and perceived exertion, found that in jobs with high workloads and high ratings for perceived exertion, the two variables are correlated, but such correlations could not be found in jobs with low workloads [43].

#### Strengths and limitations

A major strength with this study is its longitudinal study design, which allows us to draw conclusions about cause-effect relationships. The high response rate with 76% of those who answered at least one follow-up questionnaire completing all 10 monthly questionnaires is also a major strength in this study.

The fairly high incidence of neck and upper extremity symptoms could of course be debated. However, concerning shoulder and arm/hand symptoms, approximately the same figures have been reported among office workers in other studies [44,45]. In these studies the incidence rate for neck symptoms was, however, lower than in our study. The case-definition, 3 days or more during the preceding month, in our study could be questioned but we consider this cut-off to be a fairly "conservative" one in comparison with other case-definitions from the same research field and thus a strength in the study. An even more conservative definition (≥ 7 days during the preceding month) might have been appropriate, but the aim of this study to detect "early signs" of neck and upper extremity symptoms justified, in our opinion, the choice of cut-off limit. Moreover, the same cut-off has been used in other published studies from the same cohort [17,31].

A possible limitation in the study design that may have influenced the results was that the observations of working technique were made on a single occasion and within a relatively short time frame (10-15 min). Consequently, the observation did not entirely mirror the variation in working technique during a full working day. Even though all observers (ergonomists) were trained to the point that their judgments were standardized, the relatively large number of observers involved in the study might have negatively influenced inter-observer reliability, leading to an increased risk of non-dependent misclassification and thus dilution of effects. However, in a study evaluating the reliability of the checklist, using more than one ergonomist in a similar population of professional computer users, the majority of the variables included in this study showed at least fair to good intra- and inter-observer reliability [46].

Another possible limitation is that the investigated variables, perceived exertion and comfort, as well as the outcomes measurements were self-reported. The validity of self-reports has as mentioned before been questioned [34,35]. In this case when non traditional "exposures" or rather early signs more related to perceptions within the psychological dimension are used the most feasible alternative is to use self ratings. Finally, no data concerning the intensity of the pain or the effects on function due to pain were taken into consideration in this study. This means that the outcome might include participants with both severe and mild symptoms. However, between 16-18% of all cases reported reduced productivity due to neck and upper extremity symptoms according to another study within the same cohort, this, in addition, might be interpreted as a limitation in function/capability due to pain [6].

## Conclusions

There was a strong association between perceived exertion and the development of neck, shoulder, and arm/hand symptoms. Moreover, there was an association between perceived comfort during computer work and incident neck symptoms. Surveillance of computer users may include perceived exertion and comfort to target individuals at risk for neck and upper extremity symptoms.

## Competing interests

The authors declare that they have no competing interests.

## Authors' contributions

AL was part of the team that collected data for this study, participated in the design of the study, performed the data analyses and drafted the manuscript. JW participated in the design of the study, performed the data analysis and helped to draft the manuscript. RW participated in the data analysis. MH participated in the design of the study and helped to draft the manuscript. AT participated in the design of the study and helped to draft the manuscript. EWT conceived the study, participated in the design of the study and helped to draft the manuscript. All authors read and approved the final manuscript.

## Pre-publication history

The pre-publication history for this paper can be accessed here:

http://www.biomedcentral.com/1471-2474/13/38/prepub
